# Long-Term Oncologic Outcomes in Robot-Assisted and Video-Assisted Lobectomies for Non-Small Cell Lung Cancer

**DOI:** 10.3390/jcm12206609

**Published:** 2023-10-19

**Authors:** Giulia Fabbri, Federico Femia, Savvas Lampridis, Eleonora Farinelli, Alessandro Maraschi, Tom Routledge, Andrea Bille

**Affiliations:** 1 Department of Thoracic Surgery, Guy’s and St. Thomas’ NHS Trust Foundation, London SE1 9RT, UK; federico.femia@gstt.nhs.uk (F.F.); savvas.lampridis@nhs.net (S.L.); eleonora.farinelli@studio.unibo.it (E.F.); alessandro.maraschi1@nhs.net (A.M.); tom.routledge@gstt.nhs.uk (T.R.); andrea.bille@gstt.nhs.uk (A.B.); 2 AOU Città della Salute e della Scienza di Torino, University of Turin, 10124 Turin, Italy; 3 St. Orsola-Malpighi University Hospital, University of Bologna, 40126 Bologna, Italy

**Keywords:** non-small cell lung cancer (NSCLC), minimally invasive surgery, robotic surgery, long-term survival

## Abstract

This study compares long-term outcomes in patients undergoing video-assisted thoracic surgery (VATS) and robotic-assisted thoracic surgery (RATS) lobectomy for non-small cell lung cancer (NSCLC); all consecutive patients who underwent RATS or VATS lobectomy for NSCLC between July 2015 and December 2021 in our center were enrolled in a single-center prospective study. The primary outcomes were overall survival (OS), disease-free survival (DFS), and recurrence rate. The secondary outcomes were complication rate, length of hospitalization (LOS), duration of chest tubes (LOD), and number of lymph node stations harvested. A total of 619 patients treated with RATS (*n* = 403) or VATS (*n* = 216) were included in the study. There was no significant difference in OS between the RATS and VATS groups (3-year OS: 75.9% vs. 82.3%; 5-year OS: 70.5% vs. 68.5%; *p* = 0.637). There was a statistically significant difference in DFS between the RATS and VATS groups (3-year DFS: 92.4% vs. 81.2%; 5-year DFS: 90.3% vs. 77.6%; *p* < 0.001). Subgroup analysis according to the pathological stage also demonstrated a significant difference between RATS and VATS groups in DFS in stage I (3-year DFS: 94.4% vs. 88.9%; 5-year DFS: 91.8% vs. 85.2%; *p* = 0.037) and stage III disease (3-year DFS: 82.4% vs. 51.1%; 5-year DFS: 82.4% vs. 37.7%; *p* = 0.024). Moreover, in multivariable Cox regression analysis, the surgical approach was significantly associated with DFS, with an HR of 0.46 (95% CI 0.27–0.78, *p* = 0.004) for RATS compared to VATS. VATS lobectomy was associated with a significantly higher recurrence rate compared to RATS (21.8% vs. 6.2%; *p* < 0.001). LOS and LOD, as well as complication rate and in-hospital and 30-day mortality, were similar among the groups. RATS lobectomy was associated with a higher number of lymph node stations harvested compared to VATS (7 [IQR:2] vs. 5 [IQR:2]; *p* < 0.001). In conclusion, in our series, RATS lobectomy for lung cancer led to a significantly higher DFS and significantly lower recurrence rate compared to the VATS approach. RATS may allow more extensive nodal dissection, and this could translate into reduced recurrence.

## 1. Introduction

Lung cancer is the leading cause of cancer death worldwide [1]. For early-stage disease, surgical resection is currently considered the gold standard treatment. Recently, the VIOLET trial proved that the thoracoscopic minimally invasive approach is a feasible and effective approach for the surgical treatment of early-stage cancer since it is associated with improved postoperative short-term outcomes, namely less postoperative pain, fewer complications, and shorter length of hospitalization, without affecting the long-term oncologic outcomes when compared to the open approach [2]. 

In recent years, the robotic approach has been increasingly used for lung resection surgery because of its advantageous technical features, such as a three-dimensional visualization and multi-wristed instruments that allow a more precise and efficient dissection. Numerous studies have suggested that robotic surgery might be associated with similar or even better perioperative outcomes [3,4,5,6,7] compared to the thoracoscopic approach, and a higher mean number of lymph node stations harvested [4,8,9]. However, there is a lack of robust long-term oncological data for the robotic approach, and hence it is still a matter of debate whether or not robotic surgery gives any advantage in patients’ survival [9,10,11].

Therefore, the aim of our study is to compare long-term outcomes, namely overall survival (OS) and disease-free survival (DFS), and perioperative outcomes in patients who underwent video-assisted thoracic surgery (VATS) and robotic-assisted thoracic surgery (RATS) lobectomy for primary lung cancer.

## 2. Materials and Methods

### 2.1. Patient Selection

**Figure 1:**
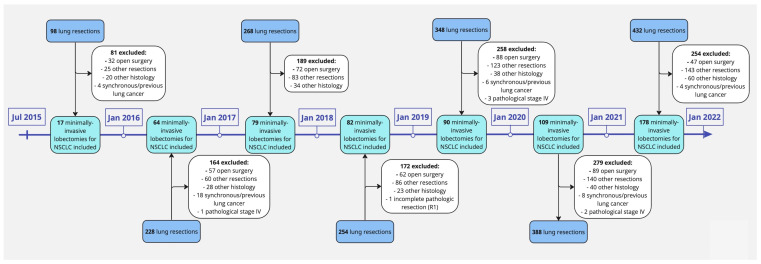
Flowchart of our lung resections’ surgical series collected between 2015 and 2021 answering to inclusion and exclusion criteria.

All consecutive patients from a prospective database who underwent minimally invasive lobectomy for non-small cell lung cancer (NSCLC) performed by two board-certified surgeons at Guy’s Hospital (Guy’s and St. Thomas’ NHS Foundation Trust, London, UK) between July 2015 and December 2021 were included in this study. Patients with other concurrent or previous primary cancers, patients with Small-Cell Lung Cancer (SCLC) or with pathological stage IV metastatic disease (according to the 8th edition of the TNM classification of malignant tumors) [12], patients without complete pathological resection, and patients who underwent a procedure other than lobectomy, namely segmentectomy, wedge resection, pneumonectomy, and chest wall resection, were excluded from the study (Figure 1).

Patients operated on before the adoption of the 8th edition of the TNM classification of malignant tumors were restaged according to the 8th edition [12]. 

Patients were characterized according to demographic variables, including age, sex, smoking history (never, former, and current smokers), clinical variables, namely performance status (<2 or ≥2), comorbidities, previous neoadjuvant therapy, forced expiratory volume in 1 s (FEV1), diffusing capacity of the lungs for carbon monoxide (DLCO), and clinical and pathological stage (I, II, III). 

Patients were divided into two groups according to surgical approach: the RATS group and the VATS group. 

### 2.2. Outcomes

The primary outcomes of this study were overall survival (OS), recurrence rate, and disease-free survival (DFS) 3 and 5 years after the surgery. A subgroup analysis comparing OS and DFS in RATS and VATS lobectomies according to the pathological stage of disease was performed. Secondary outcomes of this study were complication rate, length of hospitalization (LOS), duration of chest tubes (LOD), and number of lymph node stations harvested.

### 2.3. Follow-Up

Patients were followed up after surgery according to institutional guidelines. Follow-up visits were scheduled every 6 months for the first 2 years after surgery, then annually thereafter. At each follow-up visit, patients underwent a physical examination and thoracic computed tomography. Positron emission tomography integrated with computed tomography was performed if recurrence was suspected based on symptoms or other imaging findings. Patients were defined as lost to follow-up when they did not return for at least two consecutive follow-ups and the study team was unable to reach them.

Recurrence was defined as the presence of new lesions on imaging consistent with metastatic disease along with a biopsy confirmation if possible. Sites and dates of the first recurrence were recorded. OS was determined as the time from surgery until death from any cause or loss to follow-up. Patients who did not die during the observation period were censored at the date of the last available follow-up. DFS was defined as the time from surgery until recurrence or death from any cause. 

### 2.4. Surgical Technique

All the surgical procedures were performed by two board-certified surgeons in our center. RATS lobectomies were performed using a Da Vinci Xi Surgical Robot (Intuitive Surgical, Inc., Santa Clara, CA, USA) via 4 robotic ports (two 8 mm ports and two 12 mm ports) plus an additional port for bedside assistance and specimen retrieval. CO_2_ at a pressure of 6–8 cm H_2_O was used to perform the robotic procedure. Regarding VATS lobectomies, we used a 3-ports anterior approach according to the Copenhagen technique, as reported by Henrik J. Hansen and René H. Petersen [13].

Lymph node dissection was performed in accordance with the NCCN guidelines for NSCLC [14].

The perioperative management was similar for all the patients. We used one single postoperative drain measuring either 24 or 28 Fr. Locoregional analgesia was administered via intercostal or paravertebral blocks. 

### 2.5. Statistical Analysis

The characteristics of this study’s population are reported using numbers and percentages or median and interquartile range (IQR). Between-group differences were evaluated using the Chi-square test for categorical variables and the Wilcoxon–Mann–Whitney test for continuous variables. 

OS and DFS were estimated using the Kaplan–Meier method, with differences among groups assessed with a log-rank test and compared across groups using multivariate Cox proportional hazard models in the full cohort. 

All statistical tests were two-tailed, and *p* values < 0.05 were considered statistically significant. All analyses were carried out using GraphPad Prism version 9.5.1 (528). 

## 3. Results

### 3.1. Patients’ Characteristics

A total of 619 patients were included in the study: 403 were treated with RATS lobectomy, and 216 with VATS lobectomy. The mean age of the entire population was 70 years (±10 years), and 62.2% (*n* = 385) of the patients were women. Patient demographics, comorbidities, and tumor characteristics are listed in Table 1. The majority of patients were pathological stage I, and the predominant histologic type was adenocarcinoma in both groups. Within the RATS group, 1.5% (*n* = 6) of patients received neoadjuvant therapy, while in the VATS group, 1.9% (*n* = 4) received neoadjuvant therapy. Chemotherapy was the primary neoadjuvant treatment given, followed by combined or sequential chemo-radiotherapy. 

The average preoperative pulmonary function (DLCO percentage) was better in the RATS group than in the VATS group (76.2% vs. 71%; *p =* 0.001). For 70 patients in the RATS cohort, the respiratory function was not retrievable. 

Excluding the aforementioned differences, patients’ characteristics were similar in terms of comorbidities, respiratory function, tumor location, tumor size, histologic characteristics, pathological stage, and neoadjuvant therapy. 

### 3.2. Overall Survival

The mean follow-up period was 37 months in the whole series (29 months and 52 months for the RATS group and VATS group, respectively). Complete follow-up was achieved for all patients in the cohort, with no patients lost to follow-up. At the end of the follow-up, 481 (77.7%) patients were still alive. Out of the 138 deaths, 67 in the VATS cohort and 71 in the RATS cohort, 30 deaths were lung cancer related (14 in the RATS group and 16 in the VATS group), 11 were related to another type of cancer (6 in the RATS group and 5 in the VATS group), 81 were non-cancer related (41 in the RATS group and 40 in the VATS group), while for a total of 16 patients, the cause of death was non-retrievable (10 in the RATS group and 6 in the VATS group).

**Figure 2:**
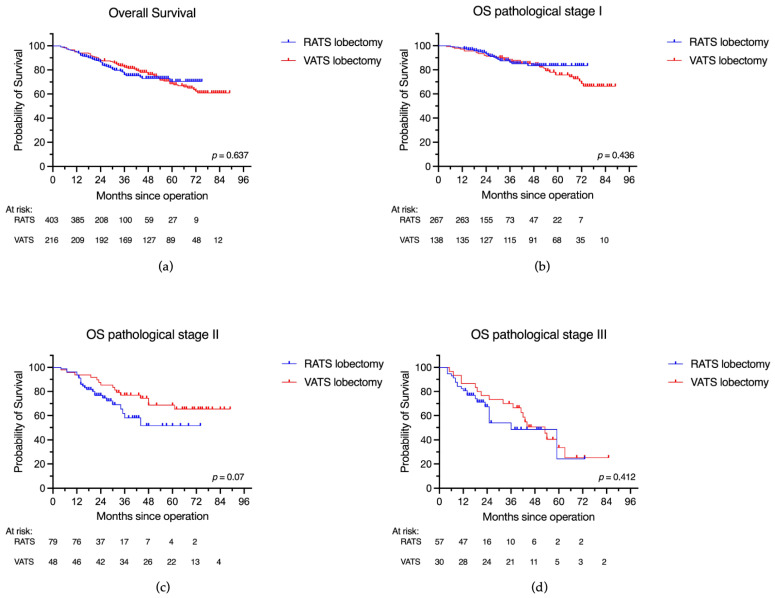
Overall survival after robotic and videothoracoscopic lobectomy for NSCLC. (**a**) Overall survival in the complete cohort; (**b**) overall survival for stage I disease after RATS and VATS lobectomy; (**c**) overall survival for stage II disease after RATS and VATS lobectomy; (**d**) overall survival for stage III disease after RATS and VATS lobectomy.

There was no statistically significant difference between RATS and VATS groups in overall survival (3-year OS: 75.9% vs. 82.3%; 5-year OS: 70.5% vs. 68.5%, respectively; *p* = 0.637). Subgroup analysis according to the pathological stage also did not show significant differences in OS between the RATS and VATS approaches (Table 2, Figure 2). These results were confirmed by the multivariate analysis, in which the surgical approach (RATS vs. VATS) was not independently associated with OS, with a hazard ratio of 1.23 (95% CI: 0.83–1.81; *p* = 0.293) for RATS compared to VATS, suggesting no significant difference in OS between RATS and VATS after adjusting for confounders. Higher pathological stage (stage II and III), as well as worse pulmonary function (DLCO), older age, and male sex, were strong predictors of worse OS, as shown in Table 3A.

### 3.3. Disease-Free Survival

**Figure 3:**
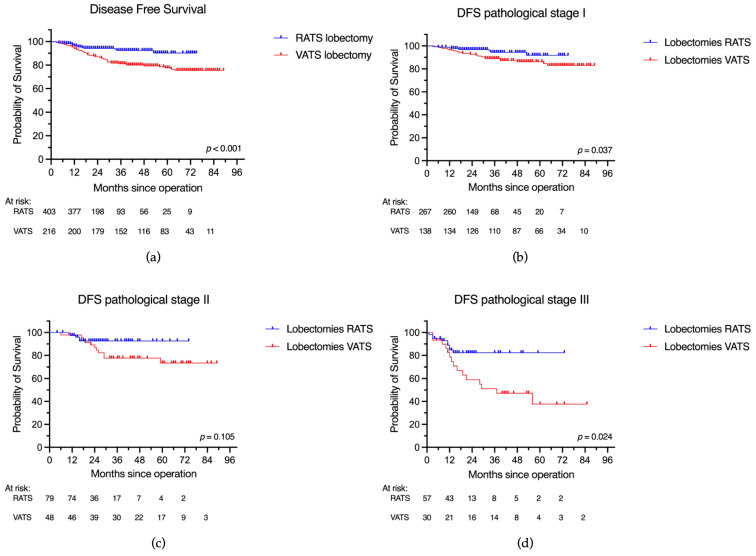
Disease-free survival after robotic and videothoracoscopic lobectomy for NSCLC. (**a**) Disease-free survival in the complete cohort; (**b**) disease-free survival for stage I disease after RATS and VATS lobectomy; (**c**) disease-free survival for stage II disease after RATS and VATS lobectomy; (**d**) disease-free survival for stage III disease after RATS and VATS lobectomy.

There was a statistically significant difference in DFS between RATS and VATS groups, favoring the robotic patients (3-year DFS: 92.4% vs. 81.2%; 5-year DFS: 90.3% vs. 77.6%, respectively; *p* < 0.001). The subgroup analysis according to the pathological stage also demonstrated a significant difference between RATS and VATS in DFS in stage I (3-year DFS: 94.4% vs. 88.9%; 5-year DFS: 91.8% vs. 85.2%, respectively; *p* = 0.037) and stage III disease (3-year DFS: 82.4% vs. 51.1%; 5-year DFS: 82.4% vs. 37.7%, respectively; *p* = 0.024). Patients in the RATS group with pathological stage II disease showed a trend of better DFS compared to those in the VATS group, but the difference was not statistically significant (3-year DFS: 92.6% vs. 77.7%; 5-year DFS: 92.6% vs. 73.4%, *p* = 0.105) (Table 2, Figure 3). In multivariable Cox regression analysis, the surgical approach (RATS vs. VATS) was significantly associated with disease-free survival, with a hazard ratio of 0.46 (95% CI 0.27–0.78; *p* = 0.004) for RATS compared to VATS. This indicates that RATS was associated with significantly better DFS compared to VATS after adjusting for other factors. Higher pathological stage (stage II and III) and male sex were significantly associated with worse DFS, as illustrated in Table 3B.

### 3.4. Recurrence Rate

A total of 72 patients (11.6%) had a recurrence, 25 (6.2%) in the RATS group and 47 (21.8%) in the VATS group, with a statistically significant difference between the groups (*p* < 0.001) (Table 4). Moreover, subgroup analysis according to pathological stage showed that patients who underwent a VATS lobectomy had a significantly higher recurrence rate in each staging group compared to RATS, as illustrated in Table 4. VATS was also associated with a significantly higher number of both local (7.4% vs. 1.2%; *p* < 0.001) and distant recurrences (11.6% vs. 4.5%; *p* = 0.001) compared to RATS.

The distant recurrences in the RATS and VATS groups occurred mainly in the brain (*n* = 17), bones (*n* = 16), contralateral lung (*n* = 8), liver (*n* = 1), adrenal gland (*n* = 2), and in the skin (*n* = 1). 

### 3.5. Surgery-Related Outcomes

Postoperative length of stay (LOS) and length of chest drain (LOD), as well as complication rate and in-hospital, 30-day, and 90-day mortality, were similar among the RATS and VATS groups (Table 5). There were no intraoperative deaths. In the RATS cohort, one patient died in hospital (0.25%) of acute respiratory distress syndrome (ARDS).

**Figure 4:**
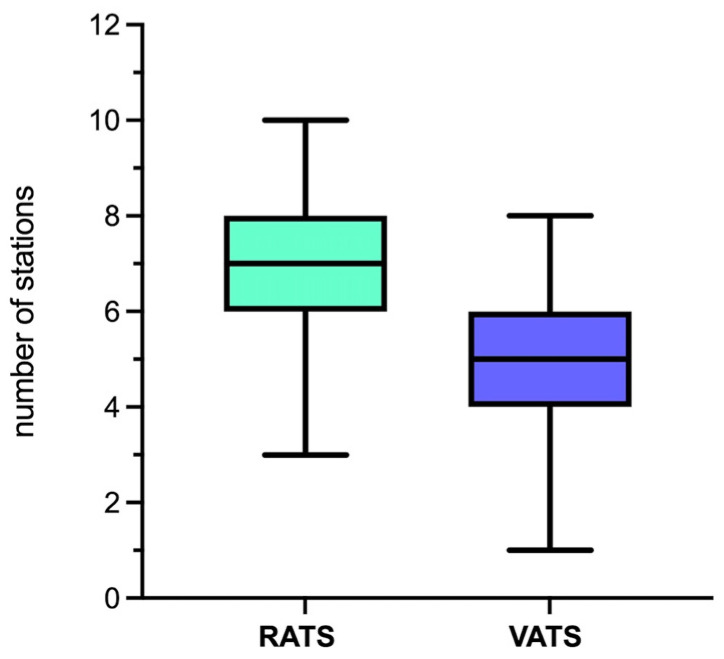
Difference in the median number of nodal stations harvested with the two approaches.

Compared to the thoracoscopic approach, the robotic approach was associated with a higher median number of lymph node stations harvested overall (5 [IQR:2] vs. 7 [IQR:2], respectively; *p* < 0.001), mediastinal (3 [IQR:1] vs. 4 [IQR:1]; *p* < 0.001) and hilar (2 [IQR:1] vs. 3 [IQR:1]; *p* < 0.001) (Table 5, Figure 4). However, the nodal upstaging rate did not differ between the RATS and VATS groups (13.2% vs. 16.7%; *p* < 0.233). There was a statistically significant difference in the upstaging rate between the RATS and VATS groups (18.6% vs. 27.8%; *p* = 0.001).

## 4. Discussion

In recent years, robotic lobectomy has been proven to be a feasible minimally invasive approach, with similar or, in some reports, even improved perioperative outcomes compared to VATS and the traditional open approach [3,4,5,6,7]. However, whether there is any difference in long-term outcomes between the robotic and thoracoscopic minimally invasive approaches is still a subject of debate.

In the current literature, there is a lack of robust data about survival comparison between robotic and VATS lobectomy in patients with NSCLC. A large propensity score study made by Kneuertz et al. [10] reported a similar locoregional and distant recurrence rate among VATS, RATS, and open lobectomy (*p* = 0.9), as well as equivalent 5-year overall survival among the groups (55%, 63%, and 65%, respectively; *p* = 0.56). A previous study made by Yang et al. [9] compared RATS, VATS, and open lobectomy with similar 5-year OS (77.6%, 73.5%, and 77.9%, respectively) and DFS (72.7%, 69%, and 65.5%, respectively), and no association was found between surgical approach and long-term survival in multivariate analysis. Our results partially support these findings, since we did not find any significant differences in OS between RATS and VATS lobectomy. However, in our series, RATS lobectomy was associated both with a significantly improved DFS and with a lower recurrence rate compared to VATS (6.2% vs. 21.8%, *p* < 0.001). In particular, the VATS approach was associated with a higher number of local recurrences compared to RATS (16 vs. 5; *p* < 0.001). Moreover, in the multivariate analysis, the RATS surgical approach was associated with significantly better DFS compared to VATS, after adjusting for other factors (HR: 0.46; 95% CI 0.27–0.78; *p* = 0.004), suggesting that there might be specific factors related to the RATS surgical approach that might confer an advantage in patients’ survival.

In several studies, the robotic approach was associated with a higher lymph node yield compared to VATS [4,8,9]. Nelson et al. [4] have reported a significantly higher mean of N2 and N1 lymph node stations collected with RATS (3.1 ± 1 and 2.5 ± 0 9, respectively) compared with both open (2.7 ± 0.9 and 1.8 ± 0.7, respectively) and VATS (2.4 ± 0.9 and 1.8 ± 0.6, respectively) (*p* < 0.001). Our findings are in line with the aforementioned results, with a median number of mediastinal and hilar lymph node stations harvested with the robotic approach of 4 [IQR:1] and 3 [IQR:1], respectively, compared with a median N2 and N1 stations collected with VATS of 3 [IQR:1] and 2 [IQR:1], respectively (*p* < 0.001). The hypothesis behind these results is that the robotic approach, due to its advantageous features, such as 3D vision, stable camera platform, and improved instrument articulation, could allow a more thorough and meticulous lymphadenectomy. The European Association of Thoracic Surgeons (ESTS) guidelines recommend a systematic nodal dissection in all cases in order to ensure a complete resection and a correct postoperative staging of the disease [15]. The International Association for the Study of Lung Cancer (IASLC)’s definition of systematic nodal dissection is the excision of ≥6 lymph nodes and ≥3 nodal stations, including the subcarinal station [16]. In several studies, a higher lymph node yield was associated with better long-term survival [17,18]; Wu et al. [19] conducted a randomized trial to investigate whether systematic nodal dissection was superior to mediastinal lymph nodal sampling in the treatment of NSCLC. They found that systematic lymphadenectomy was associated with a significantly improved 5-year survival. An adequate lymphadenectomy is essential in lung cancer surgery to obtain an accurate staging so as to identify patients who need adjuvant therapies [20]. However, in our series, the difference in the extensiveness of the robotic nodal dissection did not translate into a difference between the two approaches in nodal upstaging. The lack of difference in nodal upstaging between our series’ cohorts raises questions about whether the more extensive RATS lymphadenectomy fully explains the DFS results we obtained. One of the potential reasons for which the higher lymph node yield with RATS may contribute to better DFS, even without differences in nodal upstaging, is that the technical aspects of robotic surgery could allow for more meticulous dissection, with the removal of additional nodes not as easily reachable by VATS. This may potentially lead to the removal of micrometastatic disease, not necessarily detected in standard pathology, that could later progress to recurrence if not resected. Numerous studies have demonstrated the association between the presence of these lymph node micrometastases, detected by ancillary histopathological and molecular techniques, and a poorer OS and DFS compared to patients without nodal micrometastases, as evidenced in a systematic review and meta-analysis by Hüyük, M et al. [21]. Another meta-analysis showed that nodal micrometastases were detected in 25.3% of 2026 NSCLC cases without nodal disease in histologic examination, and that the presence of nodal micrometastases was significantly correlated with a higher recurrence rate and worse survival [22]. Considering the aforementioned evidence, the association we found in our study between RATS and both a longer DFS and a lower recurrence rate, especially of local recurrences, might be explained by a more thorough lymphadenectomy. It must be said that, in our series, there may have been underlying group differences or selection biases that favored the RATS group independently from the nodal dissection extent.

Some studies have shown that RATS is associated with less morbidity and mortality than VATS [6,7], whereas others have shown similar results among the two approaches [3,4,5]. In our series, we observed that VATS and RATS approaches were comparable in terms of LOS, LOD, complication rate, as well as in-hospital, 30-day, and 90-day mortality. These results might show that RATS is at least as feasible and safe as the video-assisted approach to perform a lobectomy.

### Strengths and Limitations of the Study

One of the main strengths of this study is the large cohort of 619 patients who underwent minimally invasive lung cancer surgery. The median follow-up time was 37 months, allowing for a robust assessment of long-term oncological outcomes, including overall survival and disease-free survival. Importantly, complete follow-up was achieved for all patients in the cohort, with no patients lost to follow-up. These complete follow-up data enhance the reliability and validity of the survival and recurrence results observed in our study sample.

The limitations of this study must also be considered. First, there might be a selection bias among the groups due to the retrospective assignment of the patients to surgical arms, and due to its single-center nature. Secondly, the lack of randomization in this study means that the two groups could differ both on measured and unmeasured factors. Finally, the length of surveillance may not be consistent among the groups. In fact, the median follow-up overall was 37 months, but the median follow-up of the patients who underwent a VATS lobectomy was almost twice as long as the one for those who had undergone a RATS lobectomy (52 months vs. 29 months, respectively). This is due to the fact that in our center, the robotic approach has been adopted since 2017, while the videothoracoscopic approach has been used since long before that. For this reason, we believe that a longer follow-up period is necessary to update the survival results in the future. Moreover, since the robotic approach was more recently adopted in our center, the RATS cohort encompasses the surgeons’ learning curve experience, whereas the VATS cohort does not, given the surgeons’ substantial prior experience of over 5 years with VATS at the study’s inception.

Despite the fact that our results are in line with similar studies published in scientific literature, large multicentric and possibly randomized trials are warranted to consolidate this evidence. 

## 5. Conclusions

In our study, robotic lobectomy for NSCLC was associated with significantly improved disease-free survival and lower recurrence rate compared to VATS, while there was no significant difference in OS between surgical approaches. RATS was associated with a higher lymph node yield compared to VATS, but there was no difference among the approaches in nodal upstaging; hence, the robotic approach may allow more extensive nodal dissection, and this could translate into a reduced recurrence rate. 

RATS and VATS showed comparable postoperative complications, hospital stay, and duration of chest drain. Our results support the continued adoption of the robotic techniques, but further studies are warranted to confirm these results and if RATS provides a durable DFS benefit over RATS. 

## Figures and Tables

**Table 1:** Patient characteristics.

Patient Characteristics	Total	VATS	RATS	*p*
Age (±SD ^1^)	70 ± 10	69 ± 10	70 ± 10	0.059
Women	62.2% (*n* = 385)	64.4% (*n* = 139)	61% (*n* = 246)	0.435
Smoking habits				
Never	14.4% (*n* = 89)	11.6% (*n* = 25)	15.9% (*n* = 64)	0.152
Former	68.8% (*n* = 426)	69.9% (*n* = 151)	68.2% (*n* = 275)	0.716
Current	16.2% (*n* = 100)	18.5% (*n* = 40)	14.9% (*n* = 60)	0.253
unknown	0.6% (*n* = 4)	0.0% (*n* = 0)	1.0% (*n* = 4)	0.304
Performance status				0.591
<2	81.1% (*n* = 502)	82.4% (*n* = 178)	80.4% (*n* = 324)	
≥2	18.9% (*n* = 117)	17.6% (*n* = 38)	19.6% (*n* = 79)	
Comorbidities				
COPD ^2^	20.7% (*n* = 128)	25.0% (*n* = 54)	18.4% (*n* = 74)	0.061
AF ^3^	6.0% (*n* = 37)	5.6% (*n* = 12)	6.2% (*n* = 25)	0.860
CAD/IHD ^4^	13.4% (*n* = 83)	14.4% (*n* = 31)	12.9% (*n* = 52)	0.622
CKD ^5^	4.2% (*n* = 26)	3.7% (*n* = 8)	4.5% (*n* = 18)	0.834
DM ^6^	11.8% (*n* = 73)	14.4% (*n* = 31)	10.4% (*n* = 42)	0.153
TIA/CVA ^7^	2.7% (*n* = 17)	1.9% (*n* = 4)	3.2% (*n* = 13)	0.441
Respiratory function				
FEV1 ^8^ (median; IQR ^9^)	91% (IQR ^9^: 30)	92% (IQR ^9^: 30)	90% (IQR ^9^: 30)	0.195
DLCO ^10^ (median; IQR ^9^)	73% (IQR ^9^: 26)	71% (IQR ^9^: 25)	76% (IQR ^9^: 26)	0.001
Tumor location				
Right				
Upper	33.6% (*n* = 208)	35.2% (*n* = 76)	32.8% (*n* = 132)	0.592
Middle	11.3% (*n* = 70)	12.1% (*n* = 26)	10.9% (*n* = 44)	0.691
Lower	21.0% (*n* = 130)	19.4% (*n* = 42)	21.8% (*n* = 88)	0.535
Left				
Upper	18.3% (*n* = 113)	17.1% (*n* = 37)	18.9% (*n* = 76)	0.663
Lower	15.8% (*n* = 98)	16.2% (*n* = 35)	15.6% (*n* = 63)	0.908
Tumor size (mean; ±SD ^1^)	28 ± 18 mm	28 ± 16 mm	29 ± 19 mm	0.979
Tumor histology				
Adenocarcinoma	76.4% (*n* = 473)	74.1% (*n* = 160)	77.7% (*n* = 313)	0.322
Squamous cell carcinoma	20.0% (*n* = 124)	21.7% (*n* = 47)	19.1% (*n* = 77)	0.461
Large cell carcinoma	3.6% (*n* = 22)	4.2% (*n* = 9)	3.2% (*n* = 13)	0.649
Clinical stage				
I	78.8% (*n* = 488)	83.8% (*n* = 181)	76.2% (*n* = 307)	0.03
II	14.6% (*n* = 90)	13.0% (*n* = 28)	15.4% (*n* = 62)	0.473
III	5.8% (*n* = 36)	3.2% (*n* = 7)	7.2% (*n* = 29)	0.048
Unknown	0.8% (*n* = 5)	0.0% (*n* = 0)	1.2% (*n* = 5)	0.169
Pathological stage				
I	65.6% (*n* = 406)	63.9% (*n* = 138)	66.5% (*n* = 268)	0.535
II	20.5% (*n* = 127)	22.2% (*n* = 48)	19.6% (*n* = 79)	0.466
III	13.9% (*n* = 86)	13.9% (*n* = 30)	13.9% (*n* = 56)	>0.999
Final nodal status				
N1	9.5% (*n* = 59)	8.3% (*n* = 18)	10.2% (*n* = 41)	0.556
N2	9.4% (*n* = 58)	10.6% (*n* = 23)	8.7% (*n* = 35)	0.470
Neoadjuvant therapy	1.6% (n = 10)	1.9% (*n* = 4)	1.5% (*n* = 6)	0.746
Chemotherapy	1.5% (*n* = 9)	1.4% (*n* = 3)	1.5% (*n* = 6)	>0.999
Chemo-radiotherapy	0.2% (*n* = 1)	0.5% (*n* = 1)	0.0% (*n* = 0)	0.349

^1^ Standard deviation; ^2^ chronic obstructive pulmonary disease; ^3^ atrial fibrillation; ^4^ coronary artery disease/ischemic heart disease; ^5^ chronic kidney disease; ^6^ diabetes mellitus; ^7^ transient ischemic attack/cerebral vascular accident; ^8^ forced expiratory volume in the 1st second; ^9^ interquartile range; ^10^ diffusing capacity of the lungs for carbon monoxide.

**Table 2:** Long-term survivals at 3 and 5 years following VATS and RATS lobectomy for NSCLC.

Survival	VATS	RATS	*p*
Overall Survival			0.637
3 years	82.3%	75.9%	
5 years	68.5%	70.5%	
OS ^1^ stage I			0.436
3 years	86.8%	86.3%	
5 years	75.7%	83.4%	
OS ^1^ stage II			0.070
3 years	77.0%	58.2%	
5 years	68.7%	51.7%	
OS ^1^ stage III			0.412
3 years	70.0%	48.5%	
5 years	33.7%	24.3%	
Disease-free Survival			<0.001
3 years	81.2%	92.4%	
5 years	77.6%	90.3%	
DFS ^2^ stage I			0.037
3 years	88.9%	94.4%	
5 years	85.2%	91.8%	
DFS ^2^ stage II			0.105
3 years	77.7%	92.6%	
5 years	73.4%	92.6%	
DFS ^2^ stage III			0.024
3 years	51.1%	82.4%	
5 years	37.7%	82.4%	

^1^ Overall survival; ^2^ disease-free survival.

**Table 3:** (A). Multivariate analysis of prognostic factors for death. (B). Multivariate analysis of prognostic factors for recurrence or death.

(A)			
Variable	HR ^1^	95% CI ^2^	*p*
Approach			
VATS	Reference	-	-
RATS	1.23	0.83–1.81	0.293
Sex			
Female	Reference	-	-
Male	1.62	1.09–2.37	0.015
Age (continuous)	1.05	1.03–1.08	<0.001
Pathology			
Adenocarcinoma	Reference	-	-
Squamous cell carcinoma	1.01	0.64–1.55	0.963
Large cell carcinoma	1.26	0.52–2.57	0.570
Pathological stage			
I	Reference	-	-
II	1.8	1.13–2.82	0.011
III	4.54	2.93–6.97	<0.001
Induction therapy			
no	Reference	-	-
yes	2.29	0.56–6.21	0.165
Comorbidities			
Pulmonary ^3^	0.99	0.64–1.50	0.960
Cardiovascular ^4^	1.12	0.76–1.66	0.572
Diabetes	1.11	0.69–1.72	0.663
Renal failure	0.36	0.11–0.89	0.051
Respiratory function			
FEV1 ^5^ (continuous)	0.99	0.99–1.00	0.168
DLCO ^6^ (continuous)	0.98	0.97–0.99	0.015
**(B)**			
**Variable**	**HR ^1^**	**95% CI ^2^**	***p***
Approach			
VATS	Reference	-	-
RATS	0.46	0.27–0.78	0.004
Sex			
Female	Reference	-	-
Male	2.02	1.20–3.37	0.008
Age (continuous)	0.99	0.96–1.01	0.205
Pathology			
Adenocarcinoma	Reference	-	-
Squamous cell carcinoma	0.48	0.22–0.96	0.052
Large cell carcinoma	0.99	0.24–2.72	0.989
Pathological stage			
I	Reference	-	-
II	2.27	1.12–4.45	0.02
III	6.44	2.82–14.12	<0.001
Induction therapy			
no	Reference	-	-
yes	1.44	0.23–4.92	0.625
Nodal upstaging			
no	Reference	-	-
yes	1.23	0.60–2.56	0.582

^1^ Hazard ratio; ^2^ 95% confidence interval; ^3^ pulmonary complications: chronic obstructive pulmonary disease, asthma, interstitial lung disease, pulmonary embolism, obstructive sleep apnea syndrome, pulmonary hypertension; ^4^ cardiovascular complications: hypertension, ischemic heart disease, atrial fibrillation, previous cardiac surgery; peripheral vascular disease; deep venous thrombosis; cerebral vascular disease; ^5^ forced expiratory volume in the 1st second; ^6^ diffusing capacity of the lungs for carbon monoxide.

**Table 4:** Recurrence rate following VATS and RATS lobectomy.

	VATS	RATS	*p*
Recurrence rate			
Overall	21.8% (*n* = 47)	6.2% (*n* = 25)	<0.001
Stage I	12.3% (*n* = 17)	4.1% (*n* = 11)	<0.001
Stage II	27.1% (*n* = 13)	6.3% (*n* = 5)	<0.001
Stage III	56.7% (*n* = 17)	15.8% (*n* = 9)	<0.001
Recurrence site			
local	7.4% (*n* = 16)	1.2% (*n* = 5)	<0.001
distant	11.6% (*n* = 25)	4.5% (*n* = 18)	0.001
both	2.8% (*n* = 6)	0.5% (*n* = 2)	0.024
Stage I			
local	3.6% (*n* = 5)	0.7% (*n* = 2)	0.048
distant	6.5% (*n* = 9)	3.0% (*n* = 8)	0.117
both	2.2% (*n* = 3)	0.4% (*n* = 1)	0.117
Stage II			
local	6.3% (*n* = 3)	0.0% (*n* = 0)	0.052
distant	16.7% (*n* = 8)	6.3% (*n* = 5)	0.075
both	4.2% (*n* = 2)	0.0% (*n* = 0)	0.141
Stage III			
local	26.7% (*n* = 8)	5.3% (*n* = 3)	0.007
distant	26.7% (*n* = 8)	8.8% (*n* = 5)	0.054
both	3.3% (*n* = 1)	1.8% (*n* = 1)	>0.999

**Table 5:** Surgery-related outcomes.

	VATS	RATS	*p*
LOS ^1^ (median; IQR ^3^)	5 days (IQR ^3^: 5)	5 days (IQR ^3^: 5)	0.453
LOD ^2^ (median; IQR ^3^)	2 days (IQR ^3^: 3)	2 days (IQR ^3^: 3)	0.818
Complication rate	38.4% (*n* = 83)	34.0% (*n* = 137)	0.291
In-hospital mortality	0.0% (*n* = 0)	0.25% (*n* = 1) *	>0.999
30-day mortality	0.0% (*n* = 0)	0.25% (*n* = 1) *	>0.999
90-day mortality	0.0% (*n* = 0)	0.25% (*n* = 1) *	>0.999
Nodal stations harvested (median; IQR)			
Overall	5 (IQR ^3^: 2)	7 (IQR ^3^: 2)	<0.001
Mediastinal (N2)	3 (IQR ^3^: 1)	4 (IQR ^3^: 1)	<0.001
Hilar or intrapulmonary (N1)	2 (IQR ^3^: 1)	3 (IQR ^3^: 1)	<0.001
Upstaging rate	27.8% (*n* = 60)	18.6% (*n* = 75)	0.001
Nodal upstaging	16.7% (*n* = 36)	13.2% (*n* = 53)	0.233
T–upstaging	26.4% (*n* = 57)	22.6% (*n* = 91)	0.322

^1^ Length of hospital stay; ^2^ length of chest drain; ^3^ interquartile range. * Patient died of hospital-acquired pneumonia and ARDS.

## Data Availability

The data presented in this study are available on request from the corresponding author. The data are not publicly available.
